# Developing a research agenda in digital workflows for the fabrication of prostheses and orthoses

**DOI:** 10.33137/cpoj.v9i2.46848

**Published:** 2026-09-09

**Authors:** J Andrysek, A.L Mayo, C.C Ngan, D Blocka, V Pendse, E Calovini, E Birley, F Azhari, C MacKay, D Dafonseca, H Daley, R Ghosh, W.C Miller, S.U Raschke, V.F Wright, J Evans, R.Z Gao, S Mirbaha, M Akhtar, S Barsky, C Bhatla, T Burton, Y Corky, P Dang, M Elshafei, I.T Garces, S Glasford, W Heim, S Kellner, B Kok, J Kooy, P Lebel, J Leith, E.D Lemaire, E Lorette, M.J Ly, S Malik, D Mazur, K Meng, M Peirone, M Poscente, B Pousett, S Ramdial, E Rossi, D Smith, J Takimoto, C Tse, J Tung, K Woodend, M Saeedi, A Eshraghi, J Zariffa, S.L Hitzig

**Affiliations:** 1. Bloorview Research Institute, Holland Bloorview Kids Rehabilitation Hospital, Toronto, Ontario, Canada.; 2. Institute of Biomedical Engineering, University of Toronto, Toronto, Ontario, Canada.; 3. Temerty Faculty of Medicine, University of Toronto, Toronto, Ontario, Canada.; 4. Sunnybrook Research Institute, Toronto, Ontario, Canada.; 5. Boundless Biomechanical Bracing, Mississauga, Ontario, Canada.; 6. Department of Mechanical & Industrial Engineering, University of Toronto, Toronto, Ontario, Canada.; 7. University Health Network, Toronto, Ontario, Canada.; 8. School of Rehabilitation Therapy, Queen’s University, Kingston, Ontario, Canada.; 9. KITE Research Institute, University Health Network, Toronto, Ontario, Canada.; 10. Department of Physical Therapy, Temerty Faculty of Medicine, University of Toronto, Toronto, Ontario, Canada.; 11. Institute of Biomedical Engineering, University of New Brunswick, Fredericton, New Brunswick, Canada.; 12. Department of Occupational Science & Occupational Therapy, Faculty of Medicine, University of British Columbia, Vancouver, British Columbia, Canada.; 13. Centre for Aging SMART Rehabilitation Research Program, Vancouver Coastal Health Institute, Vancouver, British Columbia, Canada.; 14. BCIT Applied Research, MAKE+, British Columbia Institute of Technology, Burnaby, British Columbia, Canada.; 15. Department of Physical Therapy, University of Toronto, Toronto, Ontario, Canada.; 16. Nia Technologies Inc., Toronto, Ontario, Canada.; 17. Division of Physical Medicine & Rehabilitation, Temerty Faculty of Medicine, University of Toronto, Toronto, Ontario, Canada.; 18. Hamilton Health Sciences Corporation, Hamilton, Ontario, Canada.; 19. Department of Mechanical and Aerospace Engineering, Carleton University, Ottawa, Ontario, Canada.; 20. Sunnybrook Health Sciences Centre, Toronto, Ontario, Canada.; 21. Orthotics and Prosthetics Department, Holland Bloorview Kids Rehabilitation Hospital, Toronto, Ontario, Canada.; 22. Orthotics Prosthetics Canada, Toronto, Ontario, Canada.; 23. Winnipeg Prosthetics & Orthotics, Winnipeg, Manitoba, Canada.; 24. Prosthetics and Orthotics Department, The Ottawa Hospital, Ottawa, Ontario, Canada.; 25. Department of Mechanical Engineering, University of Ottawa, Ottawa, Ontario, Canada.; 26. Anderson Orthopedics, Winnipeg, Manitoba, Canada.; 27. Victoria Hand Project, Victoria, British Columbia, Canada.; 28. Department of Clinical Neurosciences, Cumming School of Medicine, University of Calgary, Calgary, Alberta, Canada.; 29. Graduate Program in Rehabilitation Sciences, Faculty of Medicine, University of British Columbia, Vancouver, British Columbia, Canada.; 30. Barber Prosthetics Clinic, Vancouver, British Columbia, Canada.; 31. International Society for Prosthetics and Orthotics (ISPO), Copenhagen, Denmark.; 32. Department of Mechanical and Mechatronics Engineering, University of Waterloo, Waterloo, Ontario, Canada.; 33. Beacon Ortho Ltd., Saint John, New Brunswick, Canada.; 34. Systems Design Engineering, University of Waterloo, Waterloo, Ontario, Canada.; 35. Amputee Coalition of Canada, Canada.; 36. Trent University, Peterborough, Ontario, Canada.; 37. School of Physical Therapy, Department of Health and Rehabilitation Sciences, University of Western Ontario, Canada.; 38. West Park Healthcare Centre, University Health Network, Toronto, Ontario, Canada.; 39. Department of Occupational Science and Occupational Therapy, Temerty Faculty of Medicine, University of Toronto, Toronto, Ontario, Canada.

**Keywords:** Digital Workflow, 3D Printing, Prostheses, Orthoses, Modified Delphi, Prosthetics, Orthotics, Patient-centred

## Abstract

**BACKGROUND:**

Recent advancements in digital workflows, including the use of 3D printing, are transforming the prosthetics and orthotics (P&O) field. Digital technologies offer opportunities to deliver P&O services that are more efficient by reducing design and fabrication times, are less wasteful through the elimination or reduction of consumables, including plaster, foam and other materials, and are more repeatable by having digitally saved designs. Despite known advantages, the adoption of digital workflows in P&O has been inconsistent, underscoring the need for cross-sector collaboration to guide the field using evidence-informed approaches.

**OBJECTIVE:**

To collaborate with key interest groups to identify priority areas for advancing the adoption and implementation of digital workflows in the P&O field.

**METHODOLOGY:**

A modified Delphi approach was used to inform the structure of a Canada-wide initiative and involved participation of 79 representatives from key interest groups, including prosthetists, orthotists, technicians, biomedical engineers, researchers, and people with limb absence/difference. Research priorities were identified through online surveys followed by a hybrid meeting during which attendees engaged in small- and large-group discussions to refine and select priorities.

**RESULTS:**

Five key digital P&O research priorities were identified: (1) strengthening evidence on device quality and system performance compared to traditional methods; (2) developing economic and reimbursement models to support clinical integration; (3) improving education and training for digital workflows; (4) establishing standardized practice guidelines; and (5) increasing key interest group engagement to better understand user needs and perspectives. While the general components of digital workflows are now broadly understood, future efforts should focus on more specific contexts, and discussions should be more targeted and disaggregated (e.g., by device type, patient population, or care setting) and include experts with context-specific experience.

**CONCLUSION:**

This collaboration initiative provides a strategic roadmap for advancing the adoption of digital workflows in the P&O field and supports the development of national collaborations aimed at fostering innovations and evidence-based practice.

## INTRODUCTION

Prostheses and orthoses are externally applied wearable medical devices prescribed for the head, neck, trunk, and limbs as part of a comprehensive rehabilitation or treatment plan. They are typically used to enable, replace, support, or enhance movement and function in individuals with musculoskeletal, neurological, congenital, and other health conditions that affect mobility and physical performance.^1–3^ In Canada, 27% of the population, or approximately 8 million people, live with disabilities, with 1 in 10 Canadians having a condition that directly affects their mobility, and these numbers are on the rise; contributing to increasing service utilization and costs due to factors such as improved life expectancy, rising material costs, and the growing prevalence of chronic conditions associated with disability and mobility impairments.^4^ Within this broader population, in 2012, an estimated 884,540 Canadians used an orthosis or brace,^5^ and approximately 50,000 Canadians were living with limb loss,^6^ highlighting the direct relevance of prosthetic and orthotic (P&O) services to mobility-related rehabilitation needs. As key parts of physical rehabilitation, prostheses and orthoses can support greater independence and participation in daily activities, improved quality of life, and maintenance of long-term musculoskeletal health.^3,7–9^

The costs of prostheses and orthoses impact the publicly funded healthcare system across Canada. For example, Ontario’s Assistive Devices Program incurs an annual cost of over $30 million for these devices.^10^ With an aging population, demand for prostheses and orthoses and their associated health expenditures is expected to increase.^11^ Accessibility, quality, and efficiency in the delivery of assistive devices, such as prostheses and orthoses, are essential for managing rising costs and service demands.^12^ Traditional manual hand-casting methods for producing custom prostheses and orthoses are effective,^13–15^ however the fabrication step is labor-intensive and time-consuming, resulting in substantial fabrication costs and wait times for patients, families, and funding agencies.^9,16^ Hence, the time required for traditional fabrication may also limit clinicians' capacity to focus on other important aspects of patient care, including comprehensive assessment, treatment planning, patient education, and follow-up care.

Advances in digital workflows (i.e., the steps and tasks required to make entire prostheses or orthoses), including three dimensional (3D) printing, have the potential to transform P&O device fabrication by changing how devices are designed, manufactured, and delivered. In a fully digital workflow, patient anatomy is captured using optical or laser scanning, which is then modified using computer-aided design (CAD) software to incorporate clinical rectifications and biomechanical adjustments, and other patient-specific considerations needed to optimize clinical outcomes. Finalized designs can then be fabricated using additive manufacturing based on scans of the body and produced via 3D printing. These approaches can reduce reliance on labor-intensive steps like plaster casting and manual mold modifications, shorten production times, and decrease material waste.^17^ In addition, they allow for highly patient-specific designs, including variable wall thickness, lattice structures, and integrated pressure-relief or ventilation features, which may improve device fit, comfort, weight, and durability.^18,19^

Despite the potential advantages of digital fabrication, adoption within P&O clinical practice remains limited due to a range of technical, logistical, and organizational barriers. On the logistical and organizational side, costs and training requirements associated with implementing new technologies, as well as ongoing operational costs, remain poorly understood across different device types.^20^ Capital investments in equipment, such as 3D scanners and printers, along with annual software subscription fees for processing digital shape data and designing devices, can be substantial. In addition, considerable time and resources may be required to train clinicians and technicians in digital workflows, potentially reducing clinical productivity during implementation.

From a technical perspective, translating traditional workflows to digital ones presents various challenges, especially when dealing with certain devices or patient populations.^21^ For example, in traditional casting for an orthosis, clinicians can manually position and maintain a desired anatomical joint alignment during shape capture, whereas in a digital workflow, the hands, supports, or positioning devices used to hold the limb may interfere with scanning. In prostheses practice, traditional casting allows clinicians to account for the patient’s residual limb characteristics such as anatomical tissue stiffness, compressibility, and load tolerance during shape capture. In contrast, digital scanning primarily captures external geometry, requiring anatomical tissue mechanics and biomechanical considerations to be incorporated later during the design process. Scanning also requires the patient to remain temporarily immobile and, in some cases, maintain a specific limb position during image acquisition. This can be particularly challenging for certain patient populations, such as young children, individuals with spasticity or involuntary movements, and those with cognitive or behavioral impairments, potentially affecting scan quality and the accuracy of the resulting device design. While previous studies have identified a range of barriers and knowledge gaps related to the implementation of digital workflows,^20,22–24^ a clearer understanding is needed of the most significant impediments to adoption within the Canadian P&O context. Identifying and addressing these barriers will require coordinated collaboration among researchers, clinicians, industry partners, funders, persons with lived experience of needing prostheses or orthoses, and policymakers, supported by a stronger evidence base on clinical outcomes and implementation considerations.

To address this need, our team led a national initiative bringing together key interest groups across the Canadian P&O field. Through a structured, consensus-based workshop, attendees identified priority areas and actionable strategies to support the advancement and adoption of digital workflows in clinical practice, with the aim of informing a national roadmap for research, clinical implementation, and policy development in digital fabrication within Canadian P&O care.

## METHODOLOGY

The aim of this initiative was to work collaboratively with key interest groups to develop a roadmap for the use of digital workflows, including 3D printing, in the field of P&O. Our team employed a Modified Delphi Approach^25^ to provide a structure that would organize the perspectives of a diverse set of key interest group representatives.

In setting up this project, we completed the Ethics Review Assessment Tool (ER-SAT), and the project was determined to be a quality improvement project exempt from Research Ethics Board (REB) review. Participation in the survey and workshop was voluntary. All individuals who took part in the initiative were informed about the intent of developing the digital P&O priorities, as well as the dissemination of results through journal publication. All survey responses were made anonymous.

### Delphi Approach

The Delphi method is a structured group communication method for obtaining expert opinion about complex problems through a series of surveys and controlled feedback.^25^ Key features of this approach include anonymity (experts remain unidentified), iteration (the process is repeated across multiple rounds, allowing experts to reconsider their judgements), controlled feedback (the moderator summarizes the group’s previous responses), and statistical aggregation (consensus is determined by a statistical metric). Unlike traditional Delphi approaches,^26,27^ our team modified the approach to have two rounds of voting: one via an anonymous online survey and the other via an in-person meeting. The in-person session was designed to provide contextual grounding prior to re-voting; attendees first heard from invited speakers, including clinical professionals, researchers, and representatives with lived experience who shared perspectives on delivering digitally fabricated P&O devices. Attendees then reviewed the results from the first voting round and engaged in facilitated small-group discussions to further refine priorities. A critical feature of this modified approach is the intentional seating of attendees from diverse backgrounds (i.e., lived experience, regional location, professional experience, etc.) during these small group discussions to ensure diversity of perspectives, as well as the use of a neutral facilitator to manage group dynamics; both of which help to address potential biases on the topic. Given the varying levels of expertise and experience with digital workflows in P&O, our team felt it was critical to offer the Delphi panel opportunities to hear directly from others to fully develop a national roadmap that would be embraced by a diverse set of key interest groups.

Following these activities, attendees anonymously reranked their top five priorities using a real-time online voting system. This approach builds on methods previously used by our team to achieve consensus in developing research agendas across multiple domains,^28,29^ including for national research priorities^30^ and for physical activity in limb absence/difference populations.^31^ **Figure 1** provides an overview of the meeting structure.

**Figure 1:**
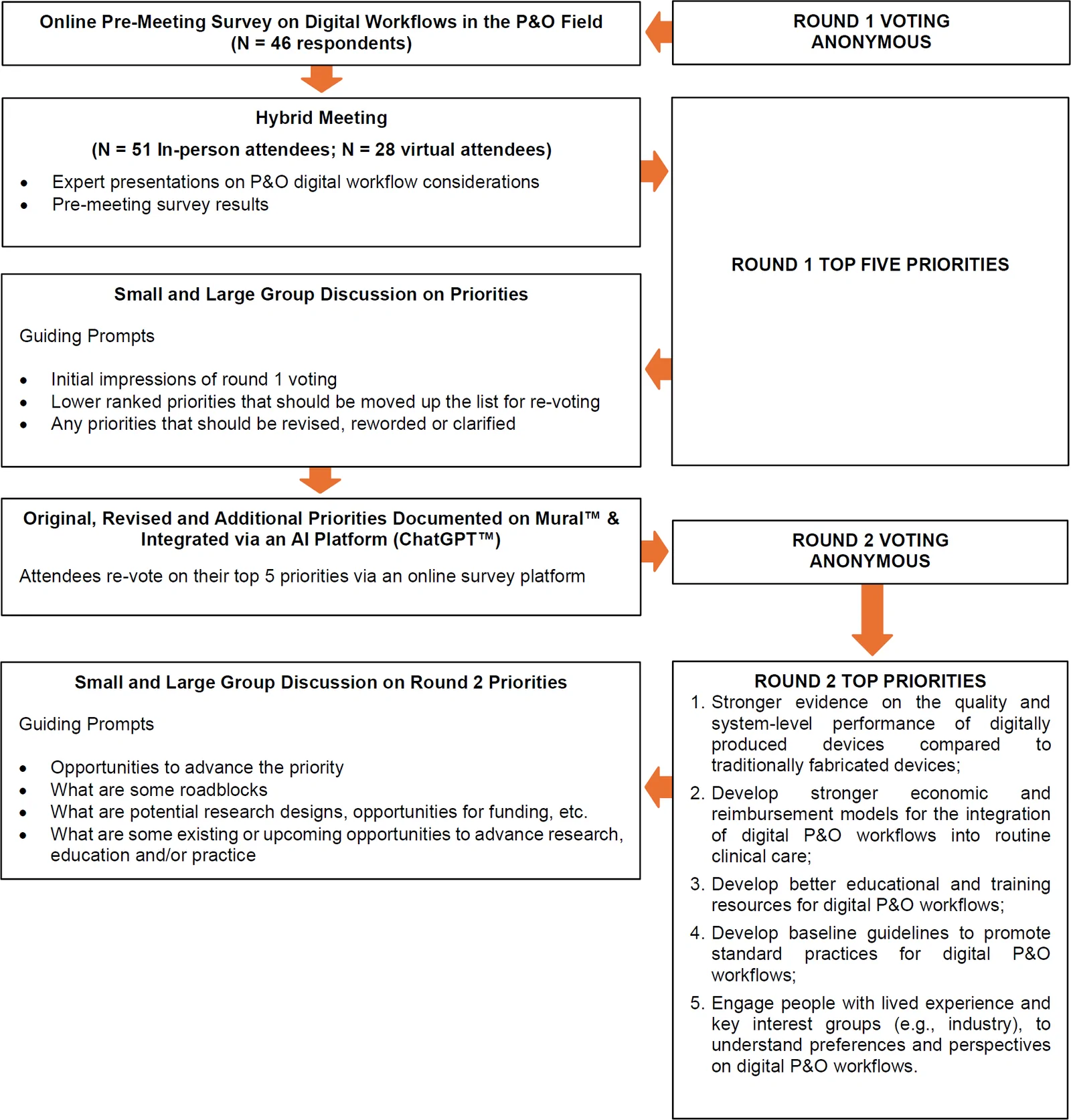
Modified Delphi structure.

### Panel Identification

To ensure the event generated a meaningful research agenda for digital workflows in P&O, our team sought to identify a diverse group of key interest groups representing lived experience, clinical practice, research, and policy perspectives. The selection of the expert panel is a critical first step in the Delphi process because it directly affects the quality and generalizability of the results. Hence, it is essential to invite individuals knowledgeable on the issue under consideration.^26,32^ Potential invitees included individuals with lived experience of needing prostheses or orthoses (e.g., persons with limb absence or limb difference), prosthetists, orthotists, technicians, biomedical and mechanical engineers, occupational therapists, physical therapists, physical medicine and rehabilitation physicians (physiatrists), researchers, students/trainees, advocacy representatives and professional organization representatives. They were identified through our team’s contacts, by reviewing the names of Canadian authors publishing on digital workflows, and through referrals from previously invited invitees. This strategy has been employed previously by our team for similar initiatives.^28,30,33^

### Pre-meeting Survey

To provide the necessary structure for obtaining consensus, the expert panel should have sufficient context and relevant information to move through the Delphi rounds.^34^ First, our core team, who organized the initiative (Andrysek J, Mayo A.L, Ngan C.C, and Hitzig S.L), all of whom have expertise in P&O, digital workflows, and 3D printing, compiled an initial list of priorities. This list was included in a pre-meeting survey (Appendix A) sent to all invitees, which asked them to select up to five top priorities from a list of ten, with the option to propose additional priorities. Survey respondents could also provide open-ended comments about digital workflows in P&O for further consideration. Additionally, the survey used a Likert scale to probe invitee agreement or disagreement, with statements reflecting attitudes toward the use of digital workflows. The invitations were sent out in May 2025, and the pre-meeting survey was open for responses between May 2025 and September 2025.

Second, prior to attending the event on October 23, 2025, which was the designated time for the second round of voting on the priorities, invitees were sent an information booklet that fully outlined the goals of the initiative, the agenda for the proposed set of activities, and the names and short biographies of each attendee (where available). Third, at the event, a set of invited speakers, including clinical professionals, researchers, and representatives with lived experience, provided an overview of key considerations and issues related to the use of digital workflows in the P&O field to help focus the discussion at the subsequent Delphi round, and each invited talk was followed by a short question and answer period. Finally, attendees were provided with the pre-meeting voting results to enable reflection and discussion of the priorities.

### Meeting Facilitation and Round Two Voting

A neutral meeting facilitator guided the discussions throughout the day. All attendees were pre-assigned to a small group, resulting in seven in-person groups and three virtual groups, each with seven to ten attendees. Efforts were made to ensure that groups were cross-sectoral (e.g., clinical, research, lived experience of using a prosthesis or orthosis, etc.). Prior to the event, the meeting facilitator provided training and materials to the core team members and supporting staff/students, who directed small group discussions over the course of the event.

The first small group discussion was designed for attendees to critically reflect on the identified priorities, consider whether lower-ranked priorities deserved further attention, and explore opportunities to revise any priorities (e.g., reword, merge priorities, etc.). All discussed priorities and modifications were captured and refined using the Mural™ Platform (Google Inc.), which was managed by a note-taker.

Following small-group discussions, each group provided a high-level overview of the key discussion points to all attendees. The initial top priorities and revisions were then imported into an online voting system from Mural™ to create a consolidated list of new priorities for review. An artificial intelligence (AI) platform (ChatGPT™) was used as a facilitation tool to summarize the complete list of priorities into a refined set for re-voting. This AI-generated summary was reviewed in real time by members of the core team to ensure that the wording was accurate and reflective of the group discussions. The final list was presented to all attendees, who then voted anonymously using an online platform (Mentimeter™).

Once the top five priorities were finalized following the re-voting, the second round of small-group discussions focused on ways to mobilize the identified research priorities into action. Opportunities for clinical and educational initiatives related to the priorities were also encouraged. The day concluded with attendees sharing insights with the larger group on ways to implement and advance priorities.

### Analysis

For the pre-meeting survey (round one), frequencies and descriptive statistics (number of attendees and device experience percentages as in **Figure 2**) were used to analyze the data. For the discussions at the workshop (round two), the collected notes on the Mural™ Platform were first narratively summarized under each of the top five identified priorities by the neutral facilitator and a member of the core team (Hitzig S.L), which were then subsequently reviewed and refined by the larger core team. This approach is reflective of deductive coding analysis,^35^ and has been applied by members of our team in past similar initiatives.^28,30,33^ All attendees were sent the manuscript to ensure the accuracy of our summary of findings and that no key viewpoints were excluded.

**Figure 2:**
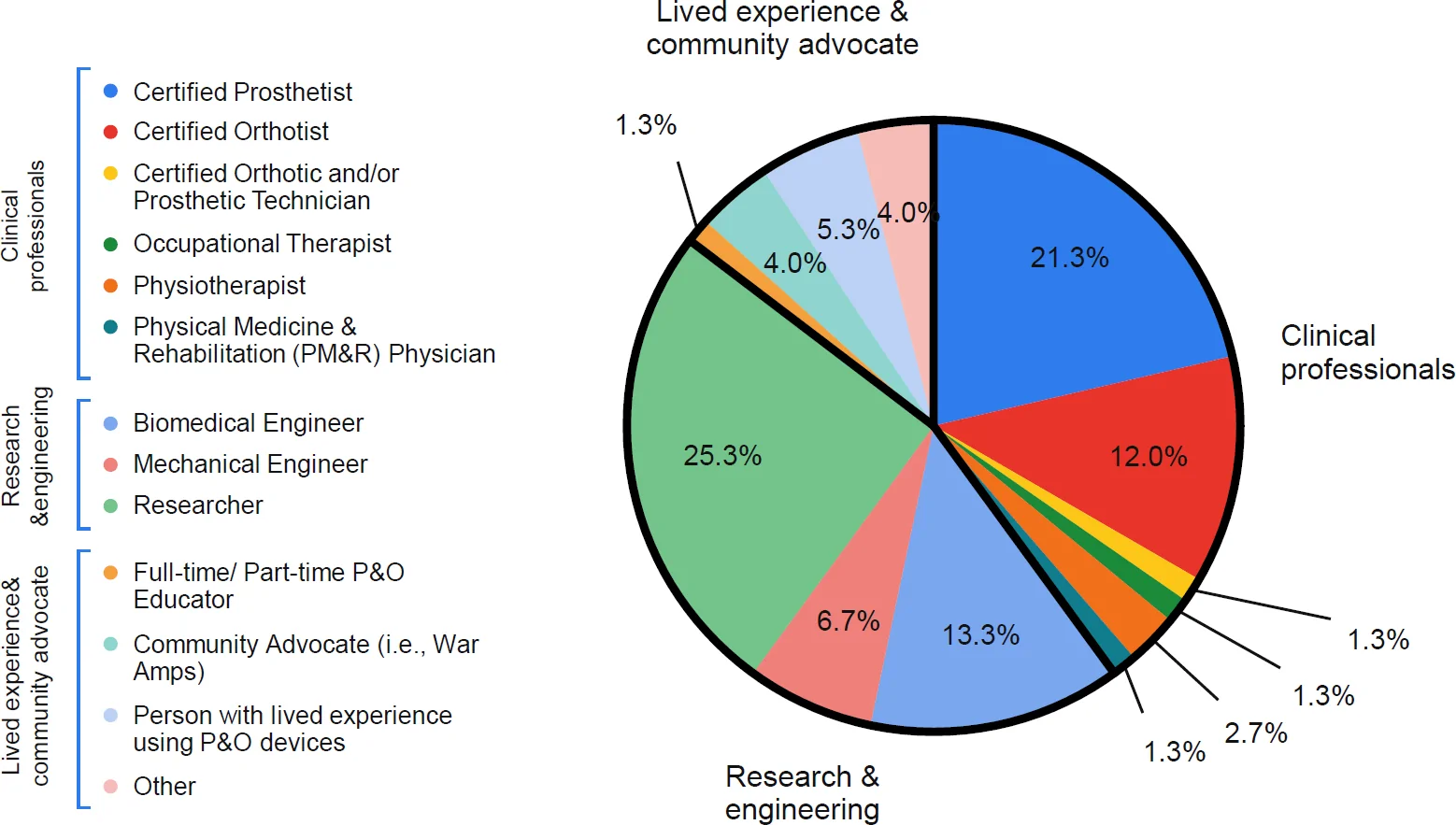
Pre-meeting survey respondents' background.

## RESULTS

In addition to the study team (n = 12) and staff/trainees (n = 9), all of whom possessed relevant expertise in digital workflows and/or P&O care through their involvement in research, development, and/or clinical practice, a cross-sectoral set of 58 invitees from across Canada were identified, for a total of 79 key interest group representatives. Forty-six invitees completed the pre-meeting survey (response rate: 58.2%). Their relevant backgrounds, with some selecting multiple applicable categories, can be seen in **Figure 3**. For the survey respondents, 46% identified as women, 54% as men, and none as other. Respondents represented five provinces across Canada, with the majority being from Ontario (71.7%), followed by British Columbia (10.9%), New Brunswick (8.7%), Alberta (8.7%), and Manitoba (8.7%).

**Figure 3:**
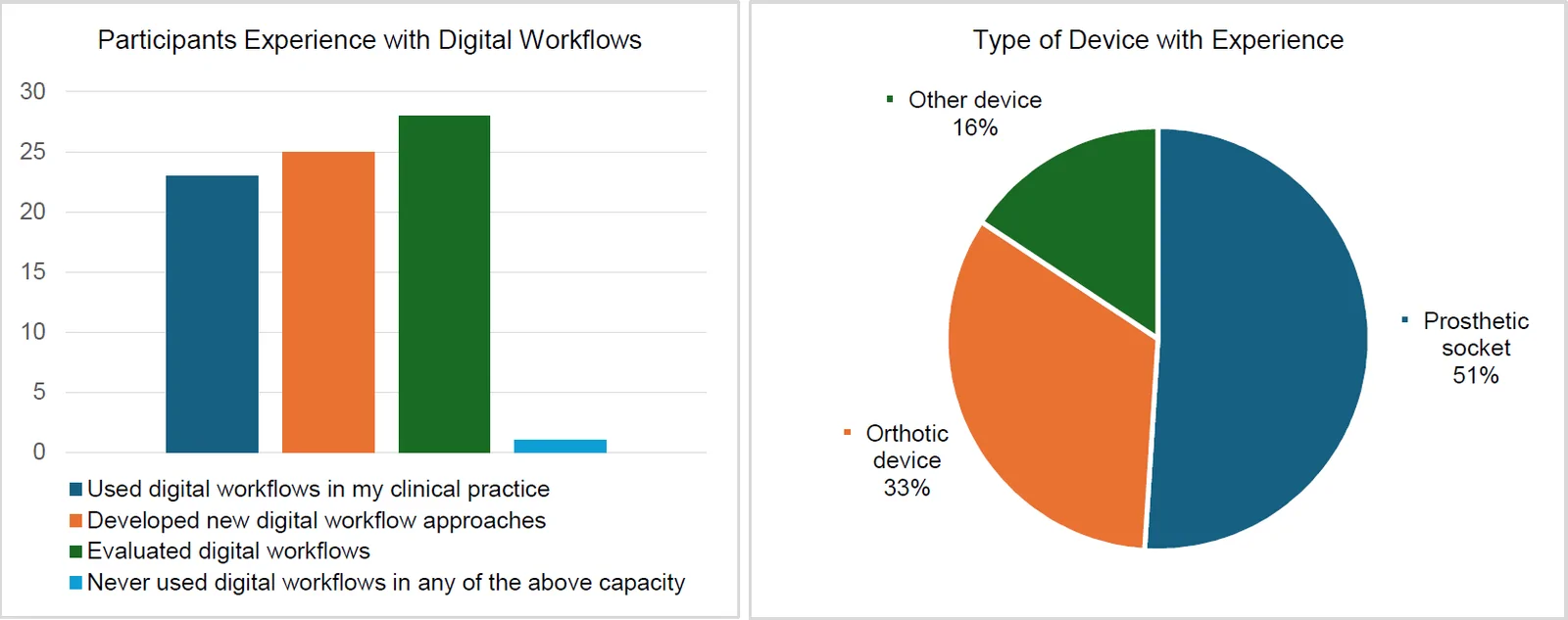
Orthotist/prosthetist survey respondents’ (n = 30) experience with developing digital technologies. The left image shows the orthotists' and prosthetists’ descriptions of their experiences with digital workflows. The right image displays the percentage of orthotists/prosthetists with experience in three device categories (orthotic devices, prosthetic sockets and other devices).

Nearly all respondents (98%) reported having some experience with digital technologies in P&O. Among them, 50% had applied digital workflows in clinical practice, 54% developed their own digital workflows or approaches to address gaps in situations where no commercially available, ready-to-use solution existed for specific prostheses or orthoses applications, and 59% had evaluated workflows through research or quality improvement initiatives. The most common applications were producing prosthetic sockets (51%) and orthotic devices (33%), with 15.7% indicating another device. Invitees’ experiences with digital workflows are summarized in **Figure 3**.

Those who had created devices using these workflows (e.g., orthotists and prosthetists) rated their satisfaction with both the i) workflow associated with designing and fabricating the device, including scanning, digital rectification, and any additional 3D modelling; and the ii) quality of the resulting device, such as aesthetics, function and strength/durability, for three device types (prostheses, orthoses, other). The satisfaction ratings are shown in **Figure 4**.

**Figure 4:**
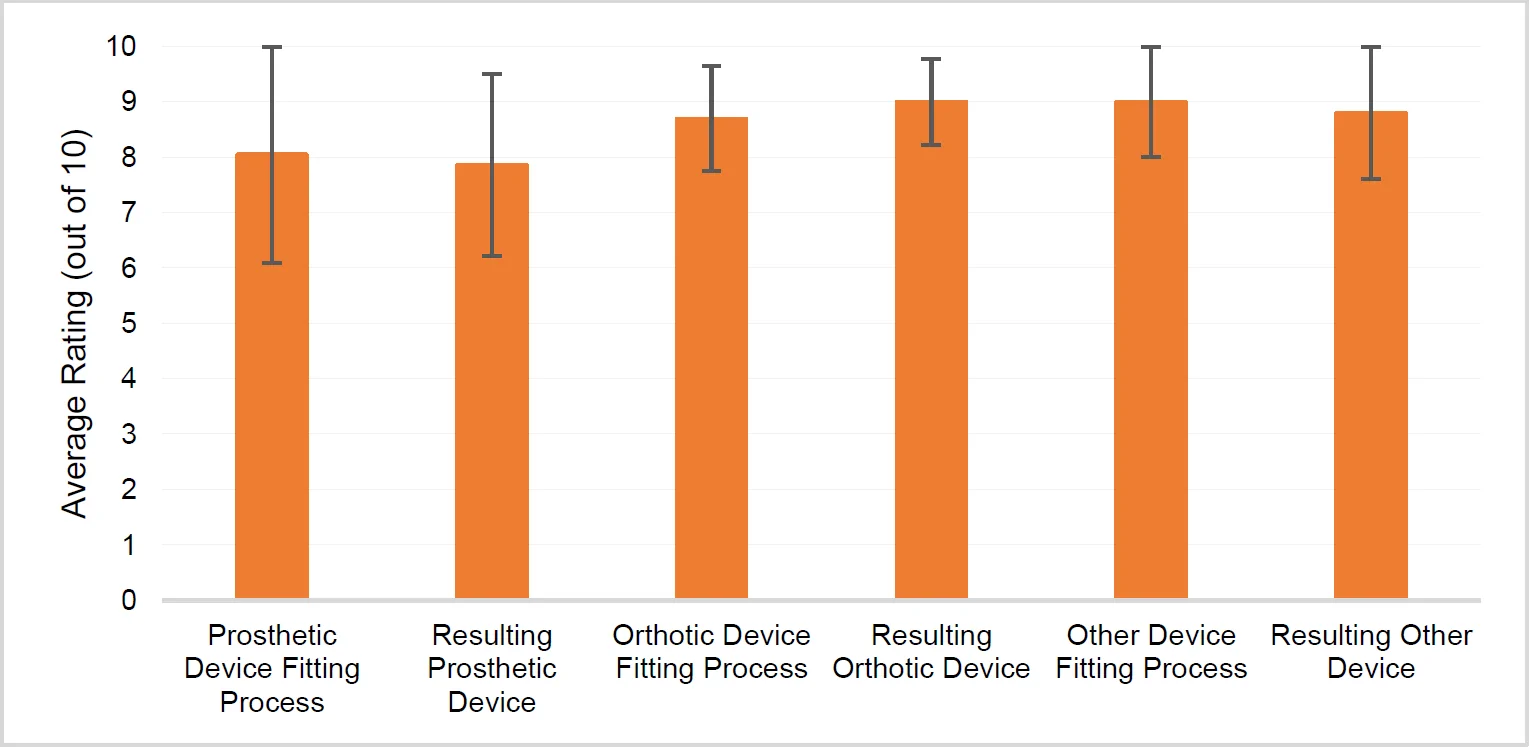
Orthotist/Prosthetist survey respondents’ (n = 30) satisfaction with digital device design and fabrication (including scanning, digital rectification, and any additional 3D modelling) and the resulting device rating (out of 10) of digital elements (mean + standard deviation [SD]).

The initial ranking of the priorities is listed in **Table 1**. Opentext responses on the survey also highlighted additional priorities not captured in the survey, including strategies to support training and culture change, clarify the role of P&O technicians, and improve accessibility for small or rural clinics. Invitees further emphasized the importance of evidence-based implementation processes, global knowledge-sharing platforms, and guidance on when digital versus traditional methods are most appropriate.

**Table 1:** List of Priorities and Initial (round 1) Rankings (n = 46). The top five ranked priorities from the initial (round 1) ranking are bolded in the table.

Research Priorities	Percentage of Responses
**Best practice guidelines to promote standard practices for digital P&O workflows**	**15.7**
**Develop better educational and training resources for digital P&O workflows**	**14.1**
**Stronger evidence on the quality of digitally produced devices compared to traditionally fabricated devices**	**13.5**
**Advance materials used in the fabrication of digital P&O devices**	**11.4**
**Develop better software to support digital P&O workflows**	**11.4**
Develop stronger economic models for integration of digital P&O workflows into routine clinical care	8.1
Develop better hardware (e.g., scanner, printers) to support digital P&O workflows	7.6
Create pathways to engage people with lived experience on their preference related to the use of digital P&O workflows	7.0
Address reimbursement policies related to digital P&O workflows	6.0
Obtain stronger evidence on the environmental impact of digital P&O workflows	4.3
Other(s)	1.1

At the hybrid workshop, the second round of anonymous voting identified the five top priorities. Specifically, three of the five top priorities remained the same, while two changed for the final overall list:

- Strengthen evidence on the quality and system-level performance of digitally produced devices compared to traditionally fabricated devices;
- Develop stronger economic and reimbursement models for the integration of digital P&O workflows into routine clinical care;
- Develop better educational and training resources for digital P&O workflows;
- Develop baseline guidelines to promote standard practices for digital P&O workflows; and
- Engage people with lived experience who use P&O devices (i.e., people with limb absence and limb difference) and key interest groups (e.g., industry), to understand preferences and perspectives on digitally made devices.

These priorities were advanced for the second round of discussion. Below, we provide a high-level summary of the key points discussed for each priority and summarize key cross-cutting issues pertinent to all the priorities.

### 1. Strengthen evidence on the quality and system-level performance of digitally produced devices compared to traditionally fabricated devices

This priority identified opportunities for designing studies and outcomes to compare traditionally and digitally fabricated prostheses and orthoses. Identified metrics included, but were not limited to, and more importantly applied variably across devices and practices: device quality (e.g., function/performance, patient preference, satisfaction, comfort, durability and strength, and device weight, as based on clinical outcomes, biomechanical performance, patient-reported outcomes etc.); efficiency (e.g., time to fit a patient with a device); frequency of modifications; environmental impact (e.g., waste); and associated costs. However, attendees cautioned that meaningful comparisons may be premature since practices related to many types of digitally fabricated devices are not as well established and refined as their traditionally fabricated counterparts. This challenge is compounded by the wide range of P&O devices and the variability in patient needs, even within a single device category. Advancing this area will also require greater consensus on definitions of quality, as well as agreement on a core set of outcome measures capable of adequately capturing and measuring quality, along with other technical and clinical outcomes that may be important.

### 2. Develop stronger economic and reimbursement models for the integration of digital P&O workflows into routine clinical care

Attendees emphasized the importance of understanding the true costs of producing digitally fabricated P&O devices. On a broader scale, engaging health economists was identified as a useful approach to comparing costs between traditional and digital production methods. Key variables to capture for such analyses include appointment duration, human resource costs, capital investments (e.g., purchase and maintenance of equipment, software, etc.), and materials. Attendees also noted the value of understanding how emerging technologies may affect third-party reimbursements, including differences between public and private third-party payers. A major challenge to advancing digital workflow adoption is the difficulty of generating a clear picture of costs, given the structural differences in funding models across provinces and territories. However, compiling existing vendor datasets and healthcare system costs, even if limited to specific regions, could provide an important foundation for strengthening the economic evidence on the potential value of digital workflows.

Other topics of discussion focused on addressing misconceptions about the costs associated with varying levels of technology quality (for example, different commercially available 3D printer options that greatly vary in purchase and operating costs, as well as the quality, strength, and appearance of produced parts) and identifying the elements required to support viable business models for organizations considering adopting a digital workflow. Attendees noted that other industry-related factors must also be mapped to better support private-sector decision-making.

### 3. Improve educational and training resources for digital P&O workflows

With the rapid growth of using digital workflow technologies in P&O, it was recognized that greater investment is needed for education on these technologies. Some expressed thoughts that education could be done in collaboration with industry. Specific lines of inquiry could focus on obtaining a better understanding of what contributes to effective P&O education and/or training on the use of digital workflows, and what training modalities might be ideal (i.e., simulation versus webinars or workshops). Opportunities to evaluate different educational models may emerge as Canadian programs transition from post-graduate diplomas to master’s-level degrees at prosthetist/orthotist training schools. At the same time, providers already in the workforce may have limited access to structured training, creating challenges in transitioning from traditional to digital methods and underscoring the need for accessible continuing professional development pathways. Attendees who had successfully implemented digital workflows in practice identified several important steps, including temporarily reducing clinical workload to allow time for learning and adaptation, visiting clinics with established workflows to gain practical insights, and dedicating a focused period of approximately two weeks to intensively adopt and integrate the technology, which was reported to improve both efficiency and quality.

### 4. Develop baseline guidelines to promote standard practices for digital P&O workflows

To advance this priority, attendees discussed the importance of segmenting the digital workflow into discrete components, such as shape capture, design, and fabrication. Within this framework, identifying a recommended pipeline for design and fabrication was suggested as a potentially valuable step. Such a pipeline would likely have components that are generalizable across devices and/or groups of patients, but likely also significant components that would be specific to a type of device, certain patient characteristics, or even the type of practice (clinic vs. hospital). Attendees also emphasized that full conversion to digital workflows may not be necessary or feasible in all contexts, and that a gradual, hybrid transition may be more appropriate in certain instances (e.g., device types). For example, as noted in the introduction, optical scanning for ankle-foot orthoses can be challenging in some patients due to difficulties maintaining the desired limb alignment during shape capture. In such cases, a hybrid workflow may involve using traditional casting to capture limb shape and alignment, followed by optical scanning of the cast and completion of the remaining design and fabrication steps using digital workflows. Attendees noted that these hybrid approaches may offer a practical pathway to adopting digital workflows by leveraging the strengths of both traditional and digital methods while accommodating clinical realities and patient-specific needs. One proposed approach for achieving this was to develop this pipeline through the aggregation of diverse design pathways into a shared database, providing clinicians with adaptable frameworks to guide decision-making. In parallel, a repository of case studies documenting the use of digital technologies and associated clinical decisions could support the development of decision-making tools or algorithms tailored to specific patient needs and device designs, thereby enhancing consistency and confidence in clinical practice.

Some workflow data collection challenges were identified. Capturing this type of data could lead to additional workload for clinicians potentially limiting the ability to gather sufficient data to meaningfully advance a practice guideline. It was also acknowledged that there might be reluctance to participate in knowledge exchange due to concerns related to proprietary rights or intellectual property arising from innovation in this space, and also privacy concerns with sharing patient data. A key motivating factor for addressing these tensions was the shared recognition of the importance of improving patient care and clinician satisfaction.

An alternative approach would be to formally learn and document the digital workflows currently in use across different clinics, using quantitative and/or qualitative research methods. Points of consideration included the following:

- Standardizing key criteria and information needed when researching and/or validating the workflows based on various hardware and software, devices being made, diagnoses/conditions, and other factors relevant to clinical practice;
- Compiling information on available technologies, including printers, software, and materials;
- Exploring applications of different technologies to reduce barriers to entry into digital workflows, such as the cost of printers for prototyping;
- Developing a recommendation guide for digital workflows including methods and hardware and software used, and a document outlining basic digital workflow literacy to support individuals new to the field; and
- Identifying barriers to digital workflow uptake by documenting where failures occurred, as well as facilitators and contributors to successful implementation and outcome evaluation.

### 5. Engage people with limb absence and limb difference and other key interest groups (e.g., professional organizations and industry), to understand preferences and perspectives on digital P&O workflows

There was agreement that advancing the field requires broad engagement across key interest groups, including persons with lived experience who use P&O devices. One proposed first step was to conduct an environmental scan (survey) to better understand who is using digital workflows and for which device types. While an initial sweeping scan could help identify key players nationally, consultations with professional organizations such as Orthotics Prosthetics Canada (OPC) and industry partners could provide a more targeted approach to help refine who should receive the survey and what information different sectors may be willing to share. A nationally recognized not-for-profit organization, such as International Society for Prosthetics and Orthotics - Canada (ISPO Canada) or OPC, could help to administer the environmental scan to reduce response biases.

### Cross-cutting Issues Across Priorities

Attendees noted that the P&O field remains somewhat siloed and that creating structured knowledge exchange opportunities such as educational modules, symposia, or research interest groups through national and international professional organizations could be an effective mechanism for fostering collaboration and a culture of continuous learning. In particular, organizations such as ISPO and OPC were again highlighted as key platforms through which such initiatives could be developed and supported.

Equity was also highlighted as an important consideration; future activities should not be focused solely on wellresourced environments, such as Canadian settings, but should be intentionally designed to be applicable across diverse global contexts. This includes not only other highincome healthcare systems, but also less-resourced settings where access to P&O services is often limited, such as rural and remote communities, disaster response contexts, and conflict-affected regions. In these environments, digital workflows may offer unique advantages, including portable scanning technologies, remote design capabilities, and decentralized or point-of-care manufacturing, but only if these approaches are adapted to local constraints such as limited infrastructure, connectivity, technical expertise, and supply chains.

Considerations related to cost, training requirements, equipment durability, and the use of locally available materials are therefore critical. Ensuring equitable implementation will require inclusive collaboration with local key interest groups, investment in capacity building, and context-sensitive research to avoid widening existing disparities and instead support more accessible and sustainable P&O service delivery globally.

Identified barriers included a lack of consistency across sites and a lack of dedicated P&O research and innovation opportunities to help in the advancement and uptake of digital workflows. Although national funding sources such as the Canadian Institutes of Health Research exist, they are highly competitive and open to a wide range of health research areas. Alternative funding approaches include partnerships with industry or government, particularly by aligning proposals with priorities such as environmental sustainability and economic development, including workforce training for emerging roles. Public engagement efforts to raise awareness of digital P&O technologies could also help stimulate investment or donations, as misconceptions about the field remain common.

Finally, as noted under the best practices priority, attendees expressed some hesitation around open knowledge exchange persisting due to proprietary and intellectual property concerns. For example, some clinics have invested substantial time and resources in developing, testing, and refining digital workflows to identify effective approaches, thereby gaining a competitive advantage that they may be reluctant to share. However, many of these concerns may be addressed through greater transparency and early dialogue among key interest groups, helping to establish clear expectations and enabling a path forward that supports increased awareness, collaboration, and broader adoption of digital workflows.

## DISCUSSION

This national initiative convened diverse key interest groups to identify priorities for advancing digital workflows for P&O in Canada. Attendees expressed optimism about the potential of digital workflows to improve efficiency, reproducibility, and patient outcomes, while also identifying gaps in evidence, education, economic evaluation, best practices, and clinical integration. It is important to note that while these themes are based on majority input from the meeting, their relevance may vary considerably depending on the context, including factors such as practice setting, experience level with digital workflows, device type, patient population, specific stages of the workflow, and the complex interactions among these elements.

From a broader perspective, digital workflows should be viewed as tools that may, in some aspects of clinical practice, replace traditional manual approaches; in others, complement them; and in some cases, remain impractical. Accordingly, a key priority for advancing their clinical adoption as identified in ***theme 1*** related to strengthening evidence is to identify where across device types, patient groups, and care settings they can most effectively improve patient care and P&O practice overall. A key priority was the need for robust comparative evidence on the quality, effectiveness, and system-level performance of digitally manufactured devices versus traditional ones. It is important to note that this need is not universal, but may be more relevant to specific device types, patient populations, or clinical applications. For example, one group reported that sufficient evidence already exists for the upper-limb prostheses they produce as part of routine care. In contrast, a pediatric rehabilitation hospital transitioning to digital workflows for ankle-foot orthoses highlighted a lack of evidence to guide clinical decision-making, particularly in determining which patients are appropriate for digital scanning versus traditional casting, such as those with more complex presentations, including spasticity. In response to these gaps, efforts are currently underway to generate evidence that will inform clinical decision-making and address these knowledge needs.

Digitization and 3D printing offer benefits like reduced material waste and production labor and time, simplified storage, expanded access (including remote and underserved regions), and advanced customization and better accommodation of patient needs, such as comfort and changes over time (i.e., children's growth).^36^ Despite the growing body of device-specific studies showing improvements in patient satisfaction, comfort, and health-related quality of life,^36–39^ uncertainty persists regarding the effectiveness of digital workflows across many types of devices and applications. Establishing a strong evidence base will require coordinated efforts to define and adopt validated clinical, functional, and economic outcome measures to ensure efficiency gains translate into meaningful patient benefits.^24,40^ Additionally, it is important to recognize that, as with traditional manual methods, much of the evidence underpinning “best” practices has historically emerged from clinical experience rather than formal research. A similar pattern is likely to occur, at least in part, in the advancement and implementation of digital methods. Therefore, it will be essential to have a critical mass of practitioners using these approaches, alongside mechanisms for sharing experiential knowledge and practical insights.

As identified within ***theme 2***, to date, evidence comparing the costs of digitally made devices with traditional ones remains limited and mixed.^41,42^ In particular, substantial upfront capital investments and ongoing operational expenses associated with digital technologies are not yet well characterized, making it difficult to draw firm conclusions about their overall economic impact. Reimbursement models further complicate adoption, as they vary widely across jurisdictions, are often governed by public payers, and are largely designed around traditional fabrication methods. Meaningful integration of digital workflows into routine clinical practice will therefore require not only stronger economic evidence, but also a restructuring of reimbursement models to reflect new modes of device production and delivery and their associated costs, ensuring financial sustainability alongside clinical innovation.^43^ At the forefront is the need for comprehensive health economic evaluations, using established health technology assessment frameworks, to inform reimbursement and support sustainable implementation across funding models.^40^

Education (***theme 3***) and the implementation of best practices (***theme 4***) were identified as key enablers of adoption, as digital workflows require specialized competencies and alignment between educational curricula and evolving clinical and industry needs, including embedded digital literacy and accessible continuing professional development.^23,44^ Establishing and integrating baseline guidelines into education and training is critical to ensure that practitioners consistently deliver high-quality care while fostering innovation. Although some global standards related to education, as well as related aspects such as products, personnel, policy and service provision exist to govern safe and accessible P&O services more broadly,^45^ research indicates that their impact and effectiveness remain poorly understood.^46^ Collaborative governance and shared resources can facilitate knowledge exchange, address proprietary concerns, and support the development of standardized guidance related to digital workflows in the field.^22,47^

Finally, ***theme 5*** relating to the meaningful engagement of individuals with lived experience requiring P&O devices, along with cross-sector key interest groups, was considered essential to ensuring patient-centered and equitable adoption. Incorporating patient perspectives not only helps to align the way devices are made with key outcomes such as comfort, usability, and accessibility, but also enables a better understanding of patient-facing gaps that these workflows can address.^48^ It is important to include a broad range of perspectives from individuals with lived experience, as access to P&O care can differ significantly across contexts, for example, between rural and urban settings. National coordination and collaboration across research, clinical, and industry sectors were identified as critical to accelerating adoption and maximizing the impact of digital technologies in P&O. A first step that attendees discussed that may be useful in achieving this goal is the use of an environmental scan, which could identify those within this space to serve as an activity to build stronger connections across sectors. Similar scans have been previously employed as a precursor to inform larger initiatives.^33,49^

This meeting and paper have several limitations. The session was conducted in a hybrid format, with in-person participation in Toronto and virtual attendance via Zoom, and required English proficiency, which may have limited broader involvement. Furthermore, the majority of all attendees were from Ontario, hence the findings may not represent all regions within Canada equally. Not every relevant key interest group may have been included, including prosthetists/orthotists during the premeeting activities, and industry partners at the meeting; as such, some perspectives may have been unintentionally overlooked. Furthermore, while many attendees had some experience with digital workflows, they were not necessarily experts in their application and as a result, the priorities identified reflect a wide range of perspectives rather than those of individuals working at the leading edge of digital workflow implementation. Also, the core team participated in the pre-meeting and meeting as part of the panel, and we have noted this in the manuscript as well as acknowledged it as a limitation. Most did not have access to information (for example, data from the pre-meeting) that might affect their perspectives on the topic. Given these considerations, along with the diversity of opinions and use cases represented, the outcomes of this meeting should not be interpreted as definitive or prescriptive recommendations, but instead are intended to serve as an initial framework to support ongoing national dialogue and collaboration aimed at strengthening capacity within the Canadian P&O sector.

Finally, in light of the limitations noted above, an important insight from this work is that while the key components of digital workflows are now becoming understood, future efforts should be more explicitly focused on specific contexts. This need is underscored by the divergent views observed across domains, for example, evidence may be considered sufficient for certain aspects of upper-limb applications but not for pediatric ankle-foot orthoses, making broad consensus difficult to achieve and potentially obscuring important areas or edge cases when relying on majority opinion. Although consensus meetings remain highly valuable, their impact could be enhanced by adopting a more targeted and disaggregated approach, either within or across meetings, such as by practice area (prostheses vs orthoses), device type, patient population (adult versus pediatric), or care setting (e.g., small clinics versus hospital systems). In parallel, it is critical to prioritize the inclusion of context-specific expertise. Defining “expert” in this setting may require consideration of individuals with direct, practical experience, including clinicians routinely delivering the digital device of interest, practitioners who have led implementation efforts, program leaders overseeing integration into clinical care, and individuals with lived experience of digitally fabricated devices.

## CONCLUSION

This initiative provides a roadmap to guide research, practice, and policy for digital workflows in P&O in Canada. The five identified priorities are strengthening evidence on device quality and system-level performance, developing economic and reimbursement models, enhancing education and training, establishing baseline guidelines, and increasing engagement of individuals with lived experience and key interest groups. Progress in these areas will require coordinated efforts across clinical, academic, industry, and policy sectors. By addressing these technical, logistical, and cultural challenges, digital workflows can be effectively integrated into routine clinical practice, supporting improved outcomes, greater efficiency, and patient-centered care. This roadmap represents a critical step toward the evidence-based, equitable, and sustainable adoption of digital technologies in the P&O field.

## Figures and Tables

**Table:** 

	Strongly Disagree	Disagree	Neutral	Agree	Strongly Agree
Digital workflows are as good as traditional approaches to P&O.	○	○	○	○	○
Digital workflows are the future of the P&O field.	○	○	○	○	○
Digital workflows require a lot more research before they should be used in routine care.	○	○	○	○	○
Digital workflows are easy to implement.	○	○	○	○	○
Digital workflows can offer clients/patients with a higher level of input into the design of their P&O device.	○	○	○	○	○

## APPENDIX 1 – Pre-Meeting Attendee Survey

**I work/live in:**

○ Newfoundland and Labrador
○ Prince Edward Island
○ Nova Scotia
○ New Brunswick
○ Quebec
○ Ontario
○ Manitoba
○ Saskatchewan
○ Alberta
○ British Columbia
○ Yukon
○ Northwest Territories
○ Nunavut
○ Outside of Canada (please specify)
○ Prefer not to answer

Outside of Canada (please specify):

___________________________________________

**Gender:**

○ Woman
○ Man
○ Non-binary/non-conforming
○ Transgender
○ Other (please specify)
○ Prefer not to answer

Other (please specify):

___________________________________________

**I am a (please select all that apply):**

□ Certified Prosthetist
□ Certified Orthotist
□ Certified Orthotic and/or Prosthetic Technician
□ Occupational Therapist
□ Physiotherapist
□ Physical Medicine & Rehabilitation (PM&R) Physician
□ Biomedical Engineer
□ Mechanical Engineer
□ Chemical Engineer
□ Researcher
□ Full-time/Part-time P&O Educator
□ Community Advocate (i.e., War Amps)
□ Person with lived experience using P&O devices
□ Student (please specify)
□ Other (please specify)
□ Prefer not to answer

Student (please specify):

___________________________________________

Other (please specify):

___________________________________________

**Which of the following fits with your occupation (please select all that apply)?**

□ I am a person with lived experience
□ I am a clinical professional
□ I work with designing prosthetic & orthotic devices using digital technology
□ Neither

**I have:**

○ Less than 1 year of experience
○ 1 to 3 years of experience
○ 3 to 5 years of experience
○ 5 to 10 years of experience
○ 10 years of experience or more
○ Prefer not to answer

**I have (please select all that apply):**

□ Upper limb amputation (including hand)
□ Partial hand or finger amputation
□ Lower limb amputation
□ Diabetic Neuropathy
□ Foot related condition (Plantar Fasciitis, Flat feet or high arches)
□ Compromise circulation (venous leg ulcers, edema, etc.)
□ Burns
□ Orthopedic condition/injury (low back pain)
□ Other (please specify):
□ Prefer not to answer

Other (please specify):

___________________________________________

**I have A PROSTHETIC DEVICE produced via a digital workflow (i.e., body part scanned, device 3D printed):**

○ No
○ Yes
○ Don't know

**I have AN ORTHOTIC DEVICE produced via a digital workflow (i.e., body part scanned, device 3D printed):**

○ No
○ Yes
○ Don't know

**I have AN OTHER DEVICE (please specify) produced via a digital workflow (i.e., body part scanned, device 3D printed):**

○ No
○ Yes
○ Don't know

Other device (please specify):

____________________________________________________

**If you received a PROSTHETIC device produced through a digital workflow, how satisfied were you with the fitting process (0 = not at all satisfied to 10 = highly satisfied).**

○ 0 ○ 1 ○ 2 ○ 3 ○ 4 ○ 5 ○ 6 ○ 7 ○ 8 ○ 9 ○ 10

If you rated your satisfaction as 5 or less, why were you not satisfied?

___________________________________________

**If you received a PROSTHETIC device produced through a digital workflow, how satisfied were you with the device (e.g., comfort, fit, use, etc.) (0 = not at all satisfied to 10 = highly satisfied).**

○ 0 ○ 1 ○ 2 ○ 3 ○ 4 ○ 5 ○ 6 ○ 7 ○ 8 ○ 9 ○ 10

If you rated your satisfaction as 5 or less, why were you not satisfied?

___________________________________________

**If you received an ORTHOTIC device produced through a digital workflow, how satisfied were you with the fitting process (0 = not at all satisfied to 10 = highly satisfied).**

○ 0 ○ 1 ○ 2 ○ 3 ○ 4 ○ 5 ○ 6 ○ 7 ○ 8 ○ 9 ○ 10

If you rated your satisfaction as 5 or less, why were you not satisfied?

___________________________________________

**If you received an ORTHOTIC device produced through a digital workflow, how satisfied were you with the device (e.g., comfort, fit, use, etc.) (0 = not at all satisfied to 10 = highly satisfied).**

○ 0 ○ 1 ○ 2 ○ 3 ○ 4 ○ 5 ○ 6 ○ 7 ○ 8 ○ 9 ○ 10

If you rated your satisfaction as 5 or less, why were you not satisfied?

___________________________________________

**If you received an OTHER device produced through a digital workflow, how satisfied were you with the fitting process (0 = not at all satisfied to 10 = highly satisfied).**

○ 0 ○ 1 ○ 2 ○ 3 ○ 4 ○ 5 ○ 6 ○ 7 ○ 8 ○ 9 ○ 10

If you rated your satisfaction as 5 or less, why were you not satisfied?

___________________________________________

**If you received an OTHER device produced through a digital workflow, how satisfied were you with the device (e.g., comfort, fit, use, etc.) (0 = not at all satisfied to 10 =highly satisfied).**

○ 0 ○ 1 ○ 2 ○ 3 ○ 4 ○ 5 ○ 6 ○ 7 ○ 8 ○ 9 ○ 10

If you rated your satisfaction as 5 or less, why were you not satisfied?

___________________________________________

**I have:**

□ Used digital workflows in my clinical practice
□ Developed new digital workflow approaches (e.g., developed software, created devices, etc.)
□ Evaluated digital workflows (e.g., research, quality improvement)
□ Never used digital workflows in any of the above capacities.
□ Other (please describe):
□ Prefer not to answer

Other (please describe):

___________________________________________

**I have not used digital workflows in my practice because (please select all that apply):**

□ I do not have access to digital workflow technologies in my practice and I am NOT interested in using them in my practice
□ I have access to digital workflow technologies but I am NOT interested in using them in my practice
□ I am interested in using digital workflow technologies in my practice but do not have access to them.
□ Other reasons for not using/interest in digital workflows (please describe)
□ Prefer not to answer

Other reasons for not using/interest in digital workflows (please describe):

___________________________________________

**What types of devices have you produced using a digital workflow (please select all that apply)?**

□ Prosthetic socket
□ Orthotic device
□ Other device (please describe)
□ Prefer not to answer

Other device (please describe):

___________________________________________

**If you have provided a PROSTHETIC device produced through a digital workflow how satisfied were you with the fitting process with your client/patient (0 = not at all satisfied to 10 = highly satisfied).**

○ 0 ○ 1 ○ 2 ○ 3 ○ 4 ○ 5 ○ 6 ○ 7 ○ 8 ○ 9 ○ 10

If you rated your satisfaction as 5 or less, why were you not satisfied?

___________________________________________

**How satisfied were you with the resulting PROSTHETIC device for your patient/client (e.g., quality, durability, etc.) (0 = not at all satisfied to 10 = highly satisfied).**

○ 0 ○ 1 ○ 2 ○ 3 ○ 4 ○ 5 ○ 6 ○ 7 ○ 8 ○ 9 ○ 10

If you rated your satisfaction as 5 or less, why were you not satisfied?

___________________________________________

**If you have provided an ORTHOTIC device produced through a digital workflow how satisfied were you with the fitting process with your client/patient (0 = not at all satisfied to 10 = highly satisfied).**

○ 0 ○ 1 ○ 2 ○ 3 ○ 4 ○ 5 ○ 6 ○ 7 ○ 8 ○ 9 ○ 10

If you rated your satisfaction as 5 or less, why were you not satisfied?

___________________________________________

**How satisfied were you with the resulting ORTHOTIC device for your patient/client (e.g., quality, durability, etc.) (0 = not at all satisfied to 10 = highly satisfied).**

○ 0 ○ 1 ○ 2 ○ 3 ○ 4 ○ 5 ○ 6 ○ 7 ○ 8 ○ 9 ○ 10

If you rated your satisfaction as 5 or less, why were you not satisfied?

___________________________________________

**If you have provided an OTHER device produced through a digital workflow how satisfied were you with the fitting process with your client/patient (0 = not at all satisfied to 10 = highly satisfied).**

○ 0 ○ 1 ○ 2 ○ 3 ○ 4 ○ 5 ○ 6 ○ 7 ○ 8 ○ 9 ○ 10

If you rated your satisfaction as 5 or less, why were you not satisfied?

___________________________________________

**How satisfied were you with the resulting OTHER device for your patient/client (e.g., quality, durability, etc.) (0 = not at all satisfied to 10 = highly satisfied).**

○ 0 ○ 1 ○ 2 ○ 3 ○ 4 ○ 5 ○ 6 ○ 7 ○ 8 ○ 9 ○ 10

If you rated your satisfaction as 5 or less, why were you not satisfied?

___________________________________________

**Please read the following statements and state how strongly you agree or disagree with them.**

**Below are a list of topics related to the use of digital workflows in the P&O field. Please review and select your top 5 priorities requiring further action to advance research, practice or policy in the P&O field. If you have an additional priority(ies), please add them to the list. Please note though you may only select 5 options**.

□ Need stronger evidence on the quality of digitally produced devices compared to traditionally fabricated devices.
□ Need best practice guidelines to promote standard practices for digital P&O workflows
□ Need to develop better educational and training resources for digital P&O workflows
□ Need to address reimbursement policies related to digital P&O workflows
□ Need to create pathways to engage people with lived experience on their preference related to the use of digital P&O workflows
□ Need to develop better software to support digital P&O workflows
□ Need to develop better hardware (e.g., scanner, printers) to support digital P&O workflows
□ Need to develop stronger economic models for integration of digital P&O workflows into routine clinical care
□ Need to advance materials used in the fabrication of digital P&O devices
□ Need to obtain stronger evidence on the environmental impact of digital P&O workflows
□ Other(s)

Other priorities:

___________________________________________

**Is there anything about digital workflows that you would like to share with us?**

___________________________________________

**Thank you for taking the time complete the survey!**

We will be hosting a full day event that will bring together clinicians, researchers, and prosthesis/orthosis users to discuss the future directions of digital workflow in P&O. We are pleased to share that the event will take place on October 23, 2025 (Thursday). It will be a hybrid event, held both in-person at Holland Bloorview Kids Rehabilitation Hospital, Toronto, and virtually via Zoom.

Would you like to attend?

○ Yes
○ No
○ Not sure yet

**When will you be able to confirm or deny your attendance?**

___________________________________________

**Why are you currently unsure of your attendance?**

___________________________________________

**Please provide if you are planning to attend in-person or virtually.**

**While efforts will be undertaken to support in-person attendance, please note that funds are limited and will be offered to invitees on a case-by-case basis.**

○ In-person
○ Virtually

**Any dietary restrictions, accessibility accommodations or any additional comments you would like us to know about?**

___________________________________________

**Please provide your name:**

___________________________________________

**Please provide your email:**

___________________________________________

**Optional please provide your phone number:**

___________________________________________

**Please recommend any others who may be a good fit for the conference (please enter their names, occupations, organizations and emails):**

___________________________________________
